# Role of Mind–Body Fitness in Obesity

**DOI:** 10.3390/diseases11010001

**Published:** 2022-12-21

**Authors:** Alexios Batrakoulis

**Affiliations:** Department of Physical Education and Sport Science, University of Thessaly, 42100 Trikala, Greece; abatrakoulis@uth.gr; Tel.: +30-24310-47018

**Keywords:** Pilates, yoga, Tai Chi, Qigong, exercise, overweight, obese, physiological adaptations, psychological adaptations

## Abstract

Various mind–body fitness modalities such as Pilates, yoga, Tai Chi, and Qigong have become an accepted part of the physical activity, exercise, and leisure sector, serving several populations eligible for meditative movement activities. However, no robust evidence is present in the current literature supporting the efficacy of these meditative movement activities on health, fitness, and well-being markers among obese adults. Interestingly, the feasibility and safety of mind–body fitness programs in this cohort are still questionable. However, the limited available data show the beneficial role of such alternative exercise options in improving numerous physical fitness and cardiometabolic health-related indicators. The major role of mind–body fitness in obese individuals is to promote muscle control, body functionality, flexibility, and balance while reducing physical limitations, chronic pain, and stress through sessions integrating body postures, efficient breathing patterns, meditation, and relaxation. Such a bodily movement-based approach may be associated with increased physical performance and improved cardiometabolic as well as mental health. However, data on anthropometric characteristics, body composition and cardiovascular disease risk factors modification are somewhat equivocal. Future studies are needed to investigate a wider spectrum of physical fitness and cardiometabolic health parameters, since obese people are likely to demonstrate poor functional capacity, impaired glucose control, lipid disorder, and abnormal blood pressure levels.

Obesity is a complex metabolic disease associated with several comorbidities, affecting almost one in three adults in the Western world, and thus, it is considered a major public health issue [1]. Notably, obese individuals are likely to show insufficient physical activity levels [2], impaired mental health [3], and poor functional capacity [4]. Taking into account that various psychophysiological health factors play a vital role in the prevention, management and treatment of obesity, physical activity, structured exercise, and eating patterns have been reported as key behavioral tools for individuals with obesity [5]. Several exercise types are currently highlighted as some of the most popular trends in the health and fitness industry worldwide [6], engaging various populations, including those with unhealthy weight. Interestingly, physical activity and exercise are highly recommended to obese individuals as a foundational piece of the treatment puzzle [7]. Specifically, traditional combined (aerobic and resistance) training appears the most beneficial exercise mode for improving a wide spectrum of cardiometabolic health parameters among obese adults [8].

However, various mind–body fitness modalities such as Pilates, yoga, Tai Chi, and Qigong are broadly used by people who seek to achieve physical and mental health outcomes [9,10,11,12,13]. In particular, meditative movement activities seem to be a feasible physical movement strategy for obese individuals, since such alternative, multicomponent modalities are not as demanding as other popular fitness trends from both a physiological and psychological standpoint [14,15]. In general, mind–body fitness focuses on body postures and movements with an emphasis on breathing techniques to promote a state of calmness and relaxation [10,16]. Hence, mind–body fitness interventions have been reported as a therapeutic or preventive exercise approach [12], demonstrating an increasing popularity in the exercise community [6,9]. However, it is not clear whether all these practices including meditative and physical elements are effective for enhancing several health, fitness, and well-being indices among obese individuals. Recent evidence shows that the most well-known and established mind–body fitness activities such as Pilates and yoga may induce positive changes in numerous psychophysiological markers in individuals with obesity [17,18]. This is an important finding considering that the majority of the world adult population belong to this cohort [1], while they do not meet the minimum recommended physical activity levels nowadays [19]. Surely, further research warrants an investigation into whether mind–body fitness is able to create a more size-inclusive exercise environment for individuals living in larger bodies.

Mind–body fitness interventions are used as alternative exercise types focusing on a group of mental, physical, and spiritual disciplines, aiming to provide a feasible and safe exercise experience to those who are previously inactive or unexperienced with these particular alternative training methods. Typically, such movement-based programs combine physical postures, breathing techniques, and sometimes meditation or relaxation aiming to enhance physical and emotional well-being. In the Western world, mind–body fitness places a lot of attention to the physical fitness aspects; however, both mental focus and spiritual energy are still fundamental components of sessions of this kind. In general, such meditative movement activities mainly emphasize on inner peace and physical energy stimulation [20]. One of the foundational training characteristics of all mind–body fitness approaches is flow, aiming to link each body posture to the appropriate breath in order to ensure flow in every pose and transition. Such an approach strategically activates the whole body without promoting muscle exhaustion. [10]. However, extreme and forceful poses should be avoided by individuals with obesity in order to reduce the risk of injury, and therefore, supervision by a qualified instructor seems to be a critical factor affecting safety and ensuring a user-friendly exercise space [21].

There is accumulating evidence showing that mind–body fitness modalities may induce positive changes in body composition [22,23,24,25], physical fitness [26,27], cardiovascular disease risk factors [13,28,29,30,31], chronic pain [32,33], and mental health [34,35] in several populations, including those affected by obesity. Hence, mind–body fitness has been defined as a component of integrated therapeutic or preventive interventions for those struggling with obesity or/and other common lifestyle-related chronic diseases [36]. Interestingly, such alternative exercise approaches appear to be effective for eliciting beneficial alterations in weight-related outcomes [22,24] in individuals with obesity. This fact is important considering the increasing popularity of mind–body fitness in the health and fitness industry, showing that regular users are primarily interested in improving health and managing body weight [37]. Given that persons with obesity commonly demonstrate physical limitations, movement dysfunctions, postural deviations, and poor stamina [4], mind–body fitness programs appear to be user-friendly and pain-free exercise strategies for this underserved population seeking for an inclusive exercise environment [38]. Figure 1 summarizes the effects of mind–body fitness interventions on cardiometabolic health, physical performance, and well-being in overweight and obese individuals [17,18,25,30,39].

Mind–body exercise interventions appear a useful addition to developing obesity management programs that focus not only on weight control but also on health and fitness benefits [12]. Pilates and yoga have been investigated more compared to Tai Chi and Qigong among individuals with obesity. Specifically, Pilates training studies primarily emphasize on body composition and physical fitness improvements. As for cardiometabolic and mental health-related markers, no robust data were reported across all trials studying these particular responses among obese persons in a free-living environment [17]. Overall, the feasibility of Pilates method as a common exercise mode for obese people is not clear. Stronger evidence is needed to identify whether this mind–body exercise modality can play an essential role in the exercise prescription guidelines for sedentary obese individuals [24]. It is worth noticing that data regarding the effectiveness of Pilates-based training programs on mental health-related indicators are currently scarce in this population.

Similarly, the feasibility of yoga practice as a mind–body fitness modality for people with unhealthy weight is still questionable, since this alternative exercise form has not been widely linked to a beneficial role in program design for obese individuals. Interestingly, yoga is commonly incorporated into an integrated lifestyle intervention as a component that promotes regular bodily movement in conjunction with healthy eating patterns, meditation, and behavior change consultation. Such an approach is not able to identify whether or not yoga alone can elicit beneficial alterations in various psychophysiological measures among obese individuals [18]. This is a common issue when examining the efficacy of yoga on health, fitness, and wellness, since yoga is not clearly considered as a type of physical exercise [36]. Thus, future research in this area should take this element into account, aiming to provide stronger evidence regarding the role of yoga in preventing, managing, and treating obesity.

With regard to Tai Chi and Qigong practice, limited data are currently available, and it is notable that these particular mind–body fitness activities are also classified as martial arts, which is something that may play a role in summarizing all relevant data from studies investigating Tai Chi and Qigong. Overall, both modalities demonstrate mixed results in various anthropometric and body composition parameters when comparing overweight/obese adults with inactive controls [25,39]. In addition, Tai Chi shows some potential since it favors positive changes in central obesity [40] and several cardiometabolic health-related indicators [13,30]. However, further robust trials are needed to provide conclusive evidence on the efficacy and safety of Tai Chi for improving major cardiovascular disease risk factors in adults with impaired metabolic health.

In summary, mind–body fitness activities such as Pilates, yoga, Tai Chi, and Qigong have not been reported as physically and mentally demanding as other conventional, but highly recommended types of exercise such as aerobic and resistance training, to overweight and obese individuals. Taking this into consideration, mind–body fitness interventions for people demonstrating poor body functionality and various physical limitations may be an attainable and pain-free exercise approach for inducing health, performance, and well-being benefits. However, further randomized controlled trials with larger samples and superior methodological quality are warranted to investigate the effects of mind–body fitness programs in individuals with obesity, aiming to identify optimal regimens that empower practitioners to interpret research findings into practical application in a real-world setting. Specifically, adherence to mind–body fitness programs as well as training configuration should be a priority in future research attempts, since compliance and dropout rates as well as training parameters such as frequency, intensity, time, and type are not well described, and in some studies, they are not even available, thus highlighting a significant gap in the current literature. This is important since mind–body fitness activities require a considerable amount of time on a regular basis (3–5 sessions/week, 60–90 min/session). Given that lack of free time has been reported as the primary exercise barrier in adults [41], while people with unhealthy weight demonstrate high attrition and low adherence to exercise [42], such a demanding weekly time commitment may not be attractive to obese individuals who seek to engage in mind–body fitness programs on a regular basis.

In conclusion, given that these meditative movement activities have been documented as alternative exercise modes frequently included in physically based lifestyle interventions with a focus on weight management and health promotion, current exercise prescription guidelines for obese individuals should take these remarks into consideration. Interestingly, meditation appears to be a common component among all mind–body fitness practices, which may play a vital role in mental health enhancement and cardiovascular risk reduction [43]. Thus, future recommendations for this population may include mind–body fitness practices as an adjunct modality, aiming to promote favorable alterations in various health, performance, and well-being aspects. However, the weak evidence and conflicting results produced by studies investigating the impact of mind–body fitness among obese persons in real-world conditions show that such alternative movement-based activities are clearly not a game changer for obesity.

## Figures and Tables

**Figure 1:**
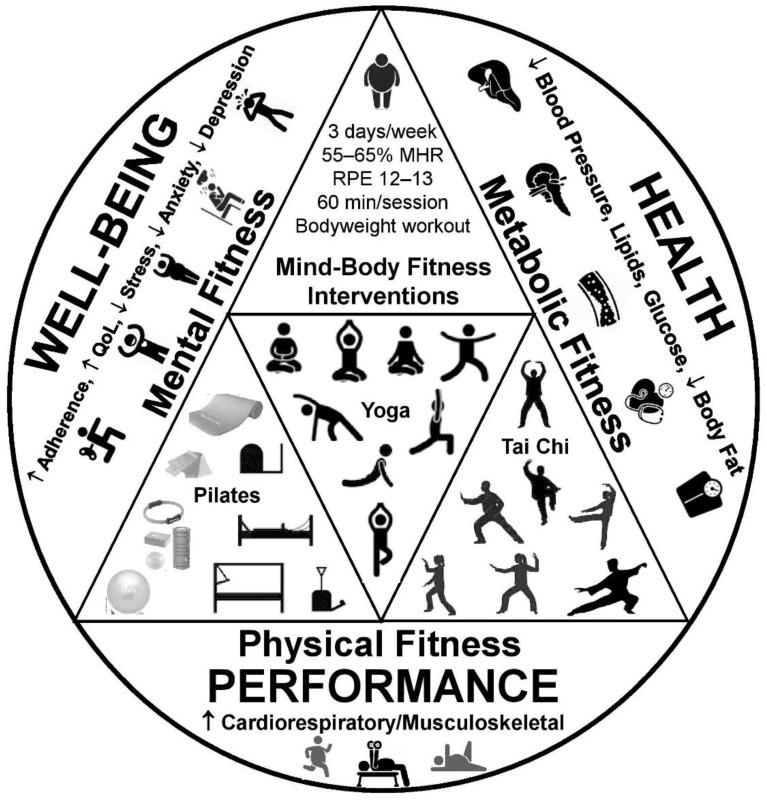
The psychophysiological effects of mind–body fitness interventions in overweight and obese individuals. MHR: maximum heart rate; QoL: quality of life; RPE: rating of perceived exertion [17,18,25,30,39].
